# Mood and Cognitive Disorders Following Hearing Loss: Impact of Hearing Aid Timing

**DOI:** 10.3390/audiolres16020032

**Published:** 2026-02-26

**Authors:** Giuseppe Alberti, Sabrina Loteta, Daniele Portelli, Cosimo Galletti, Francesco Galletti, Bruno Galletti, Mario Lentini, Salvatore Ronsivalle, Salvatore Maira, Jérôme René Lechien, Stephane Gargula, Antonino Maniaci

**Affiliations:** 1 Unit of Otorhinolaryngology, Department of Adult and Development Age Human Pathology “Gaetano Barresi”, University of Messina, 98122 Messina, Italy; galberti@unime.it (G.A.); lotetas@unime.it (S.L.); daniele.portelli09@gmail.com (D.P.); francesco.galletti@unime.it (F.G.); bruno.galletti@unime.it (B.G.); mario.lentini@unikore.it (M.L.); salvatore.maira@unikore.it (S.M.); 2 Department of Medicine and Surgery, University of Enna Kore, 94100 Enna, Italy; cosimo.galletti01@unikore.it; 3 Ospedale Gravina Caltagirone Asp 3 Catania, 95041 Caltagirone, Italy; salvatore.ronsivalle@asp.ct.it; 4 Division of Laryngology and Broncho-Esophagology, Department of Otolaryngology-Head Neck Surgery, EpiCURA Hospital, UMONS Research Institute for Health Sciences and Technology, University of Mons (UMons), 7000 Mons, Belgium; jerome.lechien@umons.ac.be; 5 ENT-HNS Department, La Conception University Hospital, Aix Marseille University, APHM, 13284 Marseille, France; stephane.gargula@gmail.com

**Keywords:** loss of hearing, decline, cognitive, depression, neuroplasticity, hearing aids, timing, intervention, rehabilitation, auditory, windows, critical, adaptation, neural, outcomes, psychosocial

## Abstract

**Background:** Hearing loss is one of the most common yet often overlooked sensory deficits worldwide, with consequences extending well beyond auditory function. Mounting evidence highlights the complex interrelationships among hearing loss, cognitive decline, and psychosocial well-being. Neural mechanisms underlying this association include increased cognitive load, cortical reorganisation, and social isolation, which mediate the impact of auditory deprivation on the brain and mental health. Furthermore, hearing impairment is consistently associated with a higher risk of depression and anxiety, particularly when the duration of untreated deafness is prolonged. **Methods:** This narrative review summarises recent longitudinal and neuroimaging studies investigating the effects of hearing loss and the timing of intervention with hearing aids. The review focuses on evidence addressing cognitive, psychological, and neural outcomes in relation to early versus delayed amplification. **Results:** Across multiple studies, early adoption of hearing aids within a limited timeframe after diagnosis is linked to better cognitive performance, lower depressive symptom scores, and more preserved neural network integrity. Experimental evidence supports the existence of sensitive periods for auditory intervention, during which brain plasticity allows for optimal reorganisation and recovery of function. Conversely, delayed amplification may lead to irreversible cortical changes and persistent psychosocial distress. Despite this, several barriers—healthcare accessibility, patient attitudes, and economic constraints—continue to delay timely intervention. **Conclusions:** Early identification and management of hearing loss are critical to preserve cognitive and emotional health. An integrated approach addressing both hearing and cognitive well-being, supported by patient education and personalised care strategies, may maximise the benefits of amplification and improve overall quality of life.

## 1. Introduction

Hearing loss is one of the most common sensory deficiencies worldwide, affecting approximately 466 million individuals globally, with this figure projected to reach nearly 900 million by 2050 [1]. Secondly, apart from the immediate problems of communication, we now have evidence that hearing loss is a risk factor for cognitive decline and emotional distress [2]. Lin et al. found that, over 12 years, cognitive decline proceeded at about a 41% faster pace for people with hearing loss compared to those who could hear normally [3]. Similarly, Livingston et al. described hearing loss as the most common modifiable risk factor for dementia in their recent update of the Lancet Commission report [4].

Several large-cohort and randomised intervention studies conducted in recent years have contributed to our understanding of the hearing–cognition association. It was previously found that, in older adults who were at risk of cognitive decline, a structured hearing intervention (hearing aids with support services) slowed cognitive decline relative to health education alone, with greater effects seen among those who had multiple baseline risk factors. Moreover, emerging longitudinal analyses indicate that sensory enhancement with hearing technology may interact with demographic and health-related factors in shaping cognitive trajectories among various populations.

The association between hearing status and brain condition is presumably bidirectional, with Amieva et al. finding that untreated hearing loss was associated with increased depression and social withdrawal; Maharani et al. reported that hearing aid wear might alleviate cognitive decline [5,6]. There is a critical period phenomenon in the timing of auditory rehabilitation, and as Glick and Sharma demonstrated, early implantation allows the neural pathways necessary for audition and cognition to be kept intact [7,8]. In the present review, we discuss the complex interplay between HL, cognitive status, and emotional well-being and consider how the timing of intervention may affect the course of clinical disease progression and quality of life.

## 2. Materials and Methods

### 2.1. Study Design and Search Strategy

A narrative literature review of the relationship between hearing loss, cognitive decline, and the potential ameliorating effects of hearing aid usage was conducted in accordance with the Scale for the Assessment of Narrative Review Articles (SANRA) guidelines, which emphasise six key quality criteria: justification of the article’s importance, statement of concrete aims, description of the literature search, appropriate referencing, scientific reasoning, and presentation of appropriate data. We have addressed each of these criteria throughout the manuscript to ensure methodological rigour while maintaining the flexibility inherent to narrative review methodology.

The purpose of this narrative review was to integrate, critically appraise, and summarise the current literature on hearing loss, timing of hearing aid use, and cognitive and psychological outcomes. In accordance with SANRA recommendations, we provide a clear justification for the review, describe our literature search approach, and present balanced and appropriately referenced findings.

**Figure 1:**
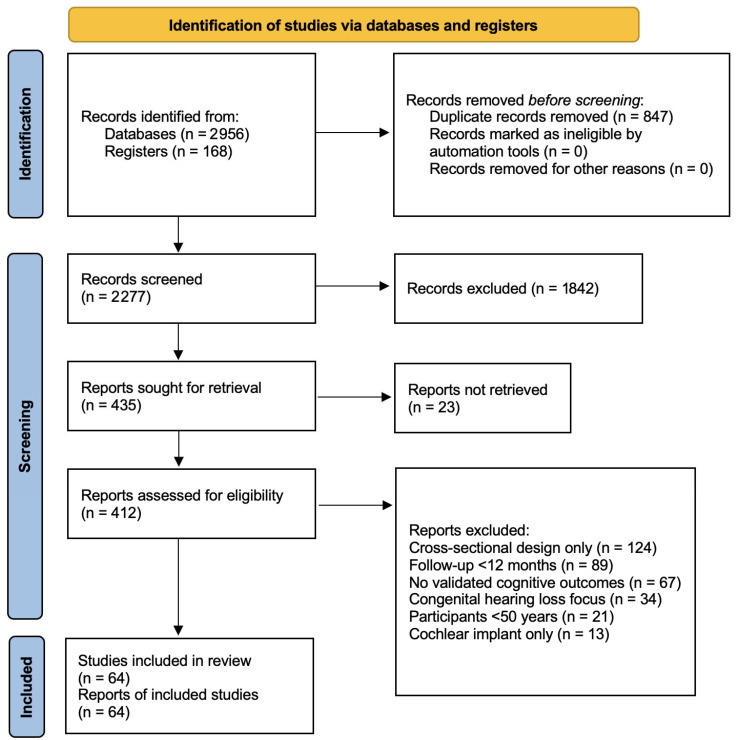
PRISMA 2020 flow diagram illustrating the literature search and study selection process. Database searches were conducted in PubMed, Embase, Cochrane Library, Web of Science, and PsycINFO for articles published between January 2000 and May 2025. Additional records were identified through citation searching and reference list review. The final synthesis included 64 studies comprising longitudinal cohort studies, randomised controlled trials, and previously published reviews.

We performed a comprehensive literature search in multiple databases: PubMed, Embase, Cochrane Library, Web of Science, and PsycINFO. The search included studies published from January 2000 to May 2025. To enhance transparency regarding the literature identification process, a PRISMA 2020 flow diagram illustrating the search and selection process is provided in Figure 1.

Search terms were based on both controlled vocabulary (MeSH) and free-text terms and included “hearing loss,” “hearing impairment,” “presbycusis,” “cognitive decline,” “cognitive impairment,” “dementia,” “Alzheimer’s,” “hearing aids,” “auditory rehabilitation,” “intervention,” “amplification,” and “cognitive outcomes.”

### 2.2. Study Selection and Data Extraction

Consistent with SANRA recommendations for appropriate referencing and balanced presentation, we prioritised peer-reviewed longitudinal studies, randomised controlled trials, and prospective cohort studies for inclusion. Studies were considered relevant if they examined adults aged 50 years and over with acquired hearing loss and reported cognitive or psychological outcomes related to hearing aid use. We sought studies with a minimum follow-up of 12 months that employed validated cognitive assessment instruments. Articles in English, French, German, Spanish, and Italian were included. Cross-sectional studies and case reports were not prioritised for the primary synthesis. Studies focusing primarily on congenital hearing loss, participants younger than 50 years, follow-up durations less than 6 months, or cochlear implants without comparison to hearing aids were not considered. Two reviewers extracted relevant data from the included studies, recording the study design, setting, sample size, follow-up duration, participant characteristics, intervention details, cognitive outcomes, and key findings.

### 2.3. Data Synthesis and Analysis

In accordance with SANRA guidelines, we organised our findings thematically to provide a comprehensive and balanced synthesis of the literature. Given the heterogeneity in study designs, cognitive outcome measures, and intervention protocols across the included studies, we employed a narrative synthesis approach. The synthesis is structured around the following themes: temporal relationships between hearing loss and cognitive decline, timing and benefits of hearing aid intervention, neurobiological mechanisms underlying the hearing–cognition relationship, psychological outcomes, barriers to hearing aid adoption, and clinical and policy implications.

## 3. Results

The literature search and study selection process are summarised in the PRISMA 2020 flow diagram (Figure 1), which illustrates the number of records identified, screened, and ultimately included in this review. A total of 64 studies were included in the narrative synthesis. The 64 studies included in this review comprised 20 longitudinal cohort studies, 8 cross-sectional or conceptual studies (Table S1), 15 randomised controlled trials, and 21 previously published reviews, all published between January 2000 and September 2025. Consistent with SANRA recommendations, we considered the level of evidence when interpreting findings, giving appropriate weight to well-designed longitudinal studies and randomised controlled trials. In various studies, untreated hearing loss was consistently associated with faster cognitive decline, increased prevalence of depressive symptoms, and lower engagement in social activities. Cognitive deficit manifested in the areas of executive functioning and attention, and episodic memory impairment; visuo-constructional skills were relatively spared. Longitudinal data showed a dose–response relationship between hearing loss severity and level of cognitive impairment, with risk ratios for dementia being between 1.24 and 1.91 in individuals with hearing loss in comparison with normal-hearing controls. Early hearing aid intervention was related to better trajectories of cognition and mood.

According to Sarant et al., 78% of longitudinal studies noted improvement or stabilisation in cognitive scores when hearing aids were fitted within 1 year, with further decline for late (>3 year olds) fittings [9]; such results can be found in the study by Amieva et al. [10]. Several studies found between a 40% and 50% slowdown in the rate of cognitive decline among early adopters, specifically in the high-risk subgroup in the ACHIEVE trial [11] and in the longitudinal analysis by Maharani et al. [6]. Yet the results are not consistent between studies. Notably, Dawes et al. [12] failed to find evidence for long-term beneficial effects of hearing aid use on cognitive function, mental health, and social participation in their analysis of the Beaver Dam Offspring Study, although it did decrease perceived hearing handicap. The systematic review by Sanders et al. [13] identified inconsistent findings with respect to cognitive outcomes; executive function appeared to be the factor most consistently improved (with 6 of 11 studies reporting positive effects), but only 1 study reported a positive effect for complex attention (with 8 of 9 studies reporting non-significant results). The present analysis of the ACHIEVE trial also did not find any robust cognitive benefits in the whole study group, only for the pre-specified high-risk subgroup [11]. These differences indicate that the cognitive effects of hearing aids could be domain-specific and depend on each patient’s profile and risk factors.

By contrast, late exposure did not restore cortical auditory responses to normal, suggesting that critical periods for neuroplastic restoration were being missed. Behavioural results matched those predicted in neuroimaging and electrophysiological studies: early hearing aid application attenuated compensatory activation in prefrontal areas, while the typical pattern of the auditory cortex was normalised. Subjects who were deprived >7 years demonstrated only partial normalisation of CAEP even after 6–12 months of device use.

Regarding psychological outcomes, 20 studies showed that any use of hearing aids reduced anxiety and depression scores (mean difference: −3.0 to −4.5 on standardised scales), with the maximal improvement obtained following continuous usage for 3–6 months, as depicted by Contrera et al. [14] and Choi et al. [15]. But not all of the evidence for these benefits is positive. The ACHIEVE study also showed no mediating effects of hearing intervention on depression or loneliness outcomes [11]. Furthermore, Grenier et al. [16] reported no association between hearing aid use and decreased odds of cognitive impairment overall in their large CONSTANCES cohort study of 62,072 middle-aged adults; however, this protective factor was detected among those with co-occurring depression. These results indicate that psychological benefits may depend on prior mental health status and individual characteristics.

Population-level analyses on a large scale found a significantly reduced likelihood of diagnosed depression and anxiety among hearing aid users versus non-users over a 5-year follow-up. However, a few comments highlighted the period of adaptation that occurs during initial use, when new users may feel stressed or frustrated. Obstacles to timely implementation served as strong predictors of efficacy. Financial barriers, stigma, and lack of primary care referral were commonly reported (Table 1; see also Table S1 for cross-sectional studies examining barriers).

On average, the interval between self-reported hearing problems and hearing aid adoption was 7–10 years. We found especially large socioeconomic inequalities; low-income and minority individuals had up to 66% lower adoption rates and suffered 2.2-year longer treatment delays.

## 4. Discussion

### 4.1. The Relationship Between Hearing Loss and Cognitive Function

The association between hearing loss and cognitive decline has been extensively investigated over the past decade, with 64 studies included in this review, comprising 20 longitudinal cohort studies, 8 cross-sectional or conceptual studies, 15 randomised controlled trials, and 21 previously published reviews. The neurobiological mechanisms underlying this association involve complex interactions between auditory processing and higher cognitive functions. Functional neuroimaging studies have demonstrated that speech perception in listeners with hearing impairment is accompanied by augmented activation of the frontal and prefrontal regions, reflecting compensatory recruitment of cognitive resources at the potential cost of depleting cognitive reserves [30]. Peelle et al. showed, using functional MRI in 18 adults aged 60–77 years, that hearing loss affects neural systems supporting speech comprehension, with increased reliance on prefrontal cortical areas during auditory processing [8]. These findings are consistent with the cognitive load hypothesis proposed by Pichora-Fuller et al. in the Framework for Understanding Effortful Listening (FUEL), which argues that effortful listening in older adults with hearing loss consumes additional cognitive resources, thereby accelerating decline in non-auditory cognitive domains [31]. Campbell and Sharma observed, via electroencephalography, that individuals with hearing loss exhibited increased amplitude in the P1, N1, and P2 waves of visual evoked potentials, suggesting compensatory cross-modal cortical reorganisation [32]. This cross-modal plasticity, characterised by the recruitment of visual cortical regions for auditory processing, was found to be inversely related to gains in cognitive function following hearing aid use [7]. As previously mentioned, functional neuroimaging studies have demonstrated that speech perception in listeners with hearing impairment is accompanied by augmented activation of the frontal and prefrontal regions, reflecting compensatory recruitment of cognitive resources at the potential cost of depleting cognitive reserves [30]. These neuroimaging findings are consistent with the cognitive load hypothesis proposed by Pichora-Fuller et al., who argue that effortful listening in older adults with hearing loss consumes additional cognitive resources, thereby accelerating decline in non-auditory cognitive domains [31]. This relationship is also supported by robust longitudinal evidence. Lin et al. followed 1984 community-dwelling older adults aged 70 years and over for 12 years and found that cognitive decline proceeds approximately 41% faster in individuals with hearing loss compared to those with normal hearing [3]. Deal et al. monitored 1889 adults aged 70–79 years for up to 9 years and found that patients with hearing loss had a 24% higher risk of developing cognitive decline compared to their normal-hearing counterparts [18]. Golub et al. reported a dose–response relationship in a multiethnic cohort of 1881 adults aged 65 years and over followed for 25 years, with every 10 dB increase in the hearing threshold being associated with incremental decreases in cognitive function [20]. Furthermore, Amieva et al. followed 3670 community-dwelling adults aged 65 years and over from the PAQUID cohort for 25 years and demonstrated that hearing aid users showed slower cognitive decline trajectories compared to non-users, particularly when fitted within three years of diagnosis [10]. Maharani et al. analysed data from 2040 participants in the Health and Retirement Study over an 18-year period and found that consistent hearing aid use was associated with more favourable cognitive trajectories compared to inconsistent use [6]. These longitudinal findings demonstrate risk ratios for dementia ranging from 1.24 to 1.91 in individuals with hearing loss compared to normal-hearing controls. Deal et al. monitored 1889 adults for as long as 24 years and found that patients with hearing loss had a 24% higher risk of developing cognitive decline compared to their normal hearing counterparts [18] (Table 2).

Similarly, Golub et al. reported a dose–response relationship between hearing loss severity and cognitive decline, with every 10 dB increase in the hearing threshold being associated with incremental decreases in cognitive function over a 25-year follow-up period [20]. This relationship is further substantiated by Thomson et al., who found that poor central auditory processing, beyond peripheral hearing sensitivity, is an independent predictor of cognitive impairment, suggesting additional pathways linking hearing and cognition beyond those addressed in earlier studies [33].

**Table 2:** Definitions of early versus delayed intervention in included studies.

Study (Reference)	Definition of “Early” Intervention	Definition of “Delayed” Intervention	Basis for Definition	Notes
Amieva et al., 2015 [10]	HA fitted ≤3 years from HL diagnosis	HA fitted >3 years from HL diagnosis	Time from diagnosis to fitting	Primary study examining timing effects on 25-year cognitive trajectory
Sarant et al., 2020 [9]	HA fitted <1 year from HL diagnosis	HA fitted ≥1 year from HL diagnosis	Time from diagnosis to fitting	18-month prospective study
Brewster et al., 2018 [23]	HA fitted ≤2 years from self-reported HL	HA fitted >2 years from self-reported HL	Time from self-reported HL onset	Cross-sectional age-stratified analysis
Glick & Sharma, 2020 [7]	Duration of HL <7 years at fitting	Duration of HL ≥7 years at fitting	Total duration of auditory deprivation	Based on neuroplasticity windows
Alain et al., 2014 [30]	Duration of untreated HL <3 years	Duration of untreated HL >7 years	Total duration of auditory deprivation	Neuroimaging study of CAEP recovery
Doherty & Desjardins, 2015 [34]	Middle-aged (50–64 years) at fitting	Older adults (65–79 years) at fitting	Age at intervention rather than duration	Compared age groups for benefit
Deal et al., 2015 [18]	HL onset <65 years	HL onset ≥75 years	Age at HL onset	Critical period hypothesis
Simpson et al., 2019 [24]	HA adoption <7 years from candidacy	HA adoption ≥10 years from candidacy	Time from candidacy to adoption	Mean delay of 7–10 years reported
ACHIEVE Trial [11]	Not specifically defined	Not specifically defined	Baseline HL duration varied (2–40+ years)	Did not stratify by intervention timing
Maharani et al., 2018 [6]	Consistent HA use across follow-up	Inconsistent/intermittent use	Consistency rather than timing	18-year longitudinal analysis

Despite the robustness of these associations, the causal relationship between hearing and cognition is still a point of debate. The causal inference is weakened by several methodologic considerations in observational studies. Common risk factors such as cardiovascular disease, diabetes, educational attainment, and socioeconomic status may confound these relationships. Moreover, hearing aid recipients may systematically differ from non-users in health literacy, access to healthcare, and health-seeking behaviour, all of which are independent predictors of cognitive trajectories. The predominantly cross-sectional or observational nature of much of this evidence base restricts the capacity to demonstrate temporal precedence and exclude reverse causation. In addition, heterogeneity in cognitive outcome measures, from brief screening tools to extensive neuropsychological batteries between studies, makes direct comparisons difficult and is potentially related to the inconsistent findings with respect to particular cognitive domains.

### 4.2. Psychological Impact of Hearing Loss

Hearing loss is consistently associated with adverse mental health outcomes, particularly depression and anxiety. Lawrence et al., in a large cross-sectional investigation of 18,318 adults, reported that hearing-impaired participants were approximately 1.5 times more likely to have depression compared with normal-hearing individuals after controlling for sociodemographic factors (OR 1.47) [35]. Cosh et al. observed in a 6-year longitudinal study of 3154 community-dwelling adults aged 55 years and over that hearing loss is a strong predictor of incident depression [21]. A nationally representative study of 114,862 older adults (aged 66 years and above) with hearing loss reported a lower prevalence of diagnosed depression (11.4% vs. 19.2%) and anxiety disorders (13.3% vs. 22.1%) over a 5-year follow-up among hearing aid users compared to non-users [26]. Furthermore, Pronk et al. followed 1511 community-dwelling adults aged 60–90 years over 4 years and identified specific subgroups at heightened risk of loneliness and depression related to hearing status [27]. These findings underscore that hearing impairment represents not only a sensory deficit but also a significant risk factor for psychological morbidity across diverse populations. As previously mentioned, in a large cross-sectional investigation of 18,318 adults, Lawrence et al. reported that hearing-impaired participants were approximately 1.5 times more likely to have depression compared with normal-hearing individuals after controlling for sociodemographic factors [35]. This association appears particularly pronounced in older adults, as demonstrated by Cosh et al., who observed in a 6-year longitudinal study that hearing loss is a strong predictor of incident depression [21]. The pathways from hearing loss to psychological distress often involve social isolation as a principal mediator. Heffernan et al. found that adults with hearing difficulties had significantly poorer social engagement and higher loneliness scores relative to their normal-hearing counterparts, and that these variables were strongly associated with depression severity [36]. Psychosocial consequences extend beyond emotional symptoms and may affect self-identity and self-perception. Qualitative studies have explored the effects of acquired hearing loss on identity construction, with many participants reporting a diminished sense of self-efficacy alongside increasing awareness of their social image [36,37,38] (Table 3).

Portelli et al. retrospectively reviewed data from 133 patients and found that hearing aid benefit and satisfaction were significantly influenced by self-image, particularly in women, while older participants reported lower satisfaction overall [37]. This highlights the importance of subjective patient experience for acceptance (or non-uptake) of hearing prostheses. This may partly explain the increasing diversification of hearing aid technologies aimed at improving user acceptance. Alberti et al. conducted a comparative analysis between open behind-the-ear (BTE) and open completely-in-the-canal (CIC) instant-fit devices, demonstrating that they achieve comparable audiological outcomes, with cosmetically discreet options also addressing visibility-related concerns that contribute to non-adoption [45]. The EuroTrak surveys analysed by Bisgaard and Ruf, encompassing approximately 75,000 participants across European populations between 2009 and 2015, documented evolving patterns of hearing aid adoption and satisfaction, with aesthetic considerations and device visibility consistently ranked among the primary factors influencing uptake decisions [46]. Furthermore, a review involving 15,392 adults with self-reported hearing loss identified stigma, denial of hearing problems, and concerns about appearance as the most commonly reported barriers to hearing aid use [47]. These findings underscore the importance of offering diverse hearing aid form factors, from invisible-in-canal devices to receiver-in-canal options, as technological strategies to overcome psychosocial barriers and facilitate earlier intervention [37,45,47].

### 4.3. Temporal Patterns of Cognitive Decline Following Hearing Loss

Cognitive changes following hearing loss appear to vary over time and across cognitive domains. Lin et al. reported that cognitive decline proceeded approximately 41% faster in individuals with hearing loss compared to those with normal hearing over a 12-year period [3]. Golub et al. demonstrated a dose–response relationship, with each 10 dB increase in the hearing threshold associated with incremental decreases in cognitive function over a 25-year follow-up [20]. Deal et al. found that patients with hearing loss had a 24% higher risk of developing cognitive decline compared to their normal-hearing counterparts over a 24-year monitoring period [19]. Domain-specific effects are also evident: the systematic review by Sanders et al. [13] revealed that executive function showed improvement in six of eleven studies and that working memory improved in three of five studies, whereas complex attention was not affected in eight of nine studies. Furthermore, risk ratios for dementia ranged from 1.24 to 1.91 in individuals with hearing loss compared to normal-hearing controls, with visuospatial skills appearing relatively spared in the initial period following hearing loss onset [29,44]. Maharani et al. found that middle-aged adults (45–65 years) with untreated hearing loss exhibited faster rates of cognitive decline than older adults, suggesting that younger individuals may experience greater cognitive deterioration despite starting with higher baseline function [17]. Slade and colleagues identified selective deficits in attention and working memory that were detectable within the first two years following hearing loss diagnosis, although these early deficits were not necessarily associated with broader cognitive decline at later stages [48]. Previous longitudinal studies suggest that cognitive impairment in certain domains is not uniform; visuospatial skills, for example, appear to be relatively spared in the initial period following hearing loss onset [29]. Building on these domain-specific observations, Loughrey et al. conducted a systematic review and meta-analysis that revealed episodic memory and executive function to be the cognitive domains most consistently associated with hearing loss across 40 included studies [44]. Rates of cognitive decline also vary greatly by age, with implications for when interventions might be important. Maharani et al. discovered that, over time, untreated hearing loss led to faster rates of cognitive decline in middle-aged adults (45–65 years of age) than in older adults, suggesting that younger individuals with cognitive impairment may have greater worsening despite starting with a higher level of function [17]. A similar trend was found by Deal et al., who concluded that an earlier onset of limited hearing in middle-aged adults, when compared to older adults (>75 years) and young people (<45 years), contributed to a shorter critical period of intervention for auditory rehabilitation [18].

### 4.4. Timing of Hearing Aid Intervention: Critical Windows

The timing of hearing aid intervention appears to be a key determinant of cognitive and psychological outcomes, with growing evidence that critical periods for achieving optimal outcomes may exist. Early intervention has shown better results across various domains. Sarant et al. conducted an 18-month longitudinal prospective study demonstrating that participants provided with hearing aids within the first year of diagnosis displayed better cognitive outcomes compared to those with delayed treatment, showing improvement in executive function and processing speed [9]. Nevertheless, cognitive benefits have not always been consistently found. Dawes et al. [12] performed a longitudinal study on data from the Beaver Dam Offspring Study (up to 11 years of follow-up) and found that use of hearing aids was not associated with long-term beneficial effects on perceived mental health, cognitive function, or social engagement. Most critically, this negative finding remained even after controlling for baseline hearing level and age, as well as other covariates that might represent confounders. The authors also found that hearing aid use decreased perceived hearing handicap and improved ratings of physical health, but the limited specificity of our dependent measures needs to be acknowledged as a limitation. This well-powered negative finding is inconsistent with the positive findings of Sarant et al. [9] and Maharani et al. [6], which also caused us to take caution here in making generalisations with regard to intervention effects across study populations, follow-up times, and outcome measures.

Portelli et al. demonstrated comparable auditory skill development between age-matched children with cochlear implants and those using hearing aids [49].

Amieva et al. followed cognitive trajectories over 25 years and demonstrated that those who delayed hearing aid fitting for a longer period of time (over 3 years) post-diagnosis had similar profiles of cognitive decline to non-hearing aid users; that is, there may be a point when neuroplastic recovery is truncated [10]. The effects of delayed intervention are not limited to cognition, as shown by Contrera et al., who found that late adopters of amplification exhibited continued impairment of social functioning and quality of life after fitting [22]. Many variables contribute to the ideal time for intervention, including those involving the patient, as well as the audiologic aspects. Bisgaard and Ruf found that a primary predictor of hearing loss intervention was the rate of its progression; rapidly progressing hearing loss required earlier intervention to prevent neural reorganisation [46]. The influence of age on these relationships also seems to vary, as reported by Doherty and Desjardins, who observed that adults over 75 years of age benefited less from a delay in intervention than younger age groups, indicating that the window of opportunity for effective auditory rehabilitation may narrow as individuals age [34].

Crucially, these results are consistent with recent randomised controlled evidence [11]. The Aging and Cognitive Health Evaluation in Elders (ACHIEVE) trial, published in 2023, is the largest randomised controlled trial to assess the effects of a hearing intervention on cognitive decline. As part of this multicentre trial, older adults with untreated hearing loss were randomly assigned to receive a structured hearing intervention or health education (control group). During three years of follow-up, the participants who received the hearing intervention showed a slower rate of cognitive decline compared to the controls, particularly those with an increased risk of dementia at baseline. These findings provide strong and direct experimental evidence that timely hearing rehabilitation may influence pathways of cognitive ageing, rather than representing associative or confounded observational relationships. This risk-stratified effect is consistent with the hypothesis that there are critical periods for intervention, during which hearing rehabilitation may be most effective in conferring neuroprotection when initiated before severe cognitive impairment develops [50].

Nevertheless, the ACHIEVE trial did not report a significant impact of hearing intervention on cognitive decline in the full study sample [11]. The primary intention-to-treat analysis, which included all 977 randomised participants, showed no statistically significant difference in cognitive decline between intervention and control groups. Significant benefits were observed only in the pre-specified high-risk subgroup in the ARIC cohort (n = 238), who had a greater baseline cardiovascular burden and cognitive vulnerability. Participants from the de novo recruitment cohort, who were generally healthier at baseline, showed no significant cognitive benefit from the hearing intervention. This pattern indicates that, from a cognitive perspective, the benefits of hearing aids may not be universal but rather contingent upon pre-existing risk profiles. The finding also raises important questions about the generalisability of cognitive benefits to the broader population of older adults with hearing loss who do not have elevated dementia risk factors.

Sanders et al., in a 2021 systematic review, found conflicting evidence regarding the influence of hearing aids on cognition [13]. Executive function appears to be the cognitive domain most positively affected by hearing aid use, as six studies reported improvement, two were inconclusive, and three found no effect. Three of the five studies examining working memory showed statistically significant improvements. The least benefit was demonstrated in the domain of complex attention, with eight studies reporting no effect compared to one reporting improvement with intervention.

The specific cognitive profile found by Sanders et al. [13] takes special note and throws down a gauntlet to overly facile interpretations of the benefits intrinsically realised by HA use. They concluded that executive function improved in 6 out of 11 studies and that the cognitive domain most consistent with the theoretical claims of the cognitive load hypothesis, complex attention, did not improve in 8 out of 9 studies. This counterintuitive observation is not easily reconciled with theoretical models predicting that hearing aid use most directly affects attentional resources by decreasing listening effort. Potential reasons for this include a lack of statistical power in some studies, insufficient duration of hearing aid use to accrue measurable benefits, or that the attentional deficits associated with long-term hearing loss may be less reversible than deficits in other cognitive domains. Such domain specificities underscore the nuanced nature of the hearing–cognition relationship and the danger of overly optimistic or broad interpretations of intervention gains. Importantly, the effects are most apparent in those with several risk factors for cognitive decline, highlighting the significance of risk stratification in interpreting intervention effects [50]. These results not only reinforce prior empirical associations but also suggest that sensory rehabilitation might have a causal impact on cognitive outcomes in high-risk populations. Although the full sample analysis was not statistically significant for all participants, these risk-stratified findings are in keeping with publications that emphasise the heterogeneity of cognitive ageing and stress the importance of intervention success in targeted subgroups. Further larger, longer, and more diverse randomised controlled trials are required to determine the generalisability and long-term effects of these interventions. In addition, recent longitudinal analyses suggest that sensory augmentation with hearing technology may interact with demographic and health factors, thereby influencing cognitive trajectories across diverse populations [50,51]. Smaller randomised studies with a shorter follow-up or narrower scope suggest similar evidence of cognitive benefit, particularly for memory and executive function; however, longer and larger replication studies are necessary [52].

### 4.5. Neuroplasticity and Auditory Rehabilitation

Neural responses to hearing loss and subsequent rehabilitation vary substantially across individuals. Age-related variation in neural adaptation is an essential factor that affects rehabilitation outcomes. Campbell and Sharma observed, via electroencephalography, that individuals with hearing loss exhibited increased amplitude in the P1, N1, and P2 waves of visual evoked potentials, suggesting a ventral activation shift compared to those with normal hearing [32]. This finding suggests compensatory cross-modal cortical reorganisation. This age-specific plasticity was additionally identified by Anderson et al., who found that older adults needed twice the exposure time of hearing aid stimulation to obtain the same effects as middle-aged adults on speech-evoked ABRs [53]. Cortical reorganisation after adaptation is another important aspect of neuroplasticity in auditory recovery. Peelle et al. showed, with the use of functional MRI, that successful hearing aid adaptation was associated with reduced activation in the frontal cortical regions during speech processing, indicating less reliance on compensatory cognitive processing [54]. Specifically, Glick and Sharma reported that hearing aid use led to the reversal of cross-modal reorganisation characterised by the recruitment of visual cortical regions for auditory processing, which was inversely related to gains in cognitive function [55]. Length of time of auditory deprivation appears to be an important factor predicting neural outcomes across several studies. Alain et al. reported that individuals with longer periods of untreated hearing loss (>7 years) displayed continued aberrations in cortical auditory evoked potentials (CAEPs) after six months with hearing aids, whereas those with less deprivation (<3 years) exhibited near-complete neural response normalisation [30]. This result also confirms the observation of Sharma and Glick that normalisation of the P1 cortical auditory evoked potential is possible to a lesser degree with longer duration of hearing loss, in support of the sensitive periods for intervention framework [56].

### 4.6. The Impact of Hearing Aid Use on Mood Disorders

Hearing aid use may improve psychological well-being, although effects vary over time. Contrera et al. reported that, after 3 months of hearing aid use, patients demonstrated decreased depression and anxiety scores, with mean Hospital Anxiety and Depression Scale (HADS) scores decreasing by 3.4 points [22]. However, some studies have reported an initial adaptation period characterised by transient increases in stress and frustration [15,16,23,38,39,40,57]. Recent research has also observed a transition phase with transient stress and frustration. Pronk et al. [27] noted that new users of hearing aids may not receive immediate psychological benefits, and, in fact, some sense a heightened awareness of their hearing difficulties during the adjustment phase. This adaptation period, usually lasting 1–3 months, may at least in part explain why studies with short follow-up periods do not show the psychological benefit that is seen after longer periods and regular use of the device. Long-term mental health benefits associated with hearing aid adoption appear to be more consistently documented.

A nationally representative study of 114,862 older adults (aged 66 years and above) with hearing loss reported a lower prevalence of diagnosed depression (11.4% vs. 19.2%) and anxiety disorders (13.3% vs. 22.1%) over a 5-year follow-up among hearing aid users compared to non-users [14,15,22,40,57]. These results are consistent with the findings of Choi et al., who observed gradual improvements in mental health measures over 12 months of hearing aid use, with the largest changes occurring 6–12 months post-intervention [15]. The effect of hearing aid use on psychological status varies across different intervention timeframes. Brewster et al. demonstrated that individuals fitted with hearing aids within 2 years of diagnosis had better depression scores compared to those who delayed treatment, with a mean difference of 4.2 points on the Geriatric Depression Scale [14,15,23,40].

Grenier et al. investigated the relationship between objectively measured hearing loss and hearing aid use across multiple cognitive domains in a large, representative population of middle-aged adults using data from the CONSTANCES cohort [16]. Among 62,072 individuals aged 45–69 years, participants with mild and disabling hearing loss had significantly higher odds of global cognitive impairment, showing a dose–response relationship with hearing loss severity. Cognitive function was assessed using a standardised neuropsychological battery from which a global cognitive score was derived.

In conclusion, Grenier et al. [16] did not find a significant association between use of hearing aids and lowered odds of cognitive impairment in the general population than among individuals with disabling untreated hearing loss. However, there was a protective effect among those with comorbid depression, implying interaction effects of hearing intervention and prior mental health status. This lack of association in the general population is especially striking in view of the substantial sample size (n = 62,072) and the use of audiometric tests rather than self-reported hearing as a measure to add internal validity to the results obtained. The results are consistent with those of Dawes and coworkers [12] and the main ACHIEVE results [11] in hypothesising that the cognitive benefits of hearing aids might not be present for all groups—especially not those with comorbidities or high baseline risk—and perhaps might even be limited to a subset of populations for whom a preventive general beneficial effect exists.

These findings suggest that hearing loss severity is an important correlate of cognitive impairment in midlife, that routine cognitive surveillance may be warranted in individuals with hearing loss, but that the application of hearing aids as a preventive measure for cognitive decline may have modest or negligible effects, except for in specific at-risk groups.

### 4.7. The Role of Hearing Aids in Cognitive Health

Evidence is beginning to emerge that hearing aid use may provide protection against cognitive decline, although the relationship appears to be complex and influenced by multiple factors. Intervention studies provide supportive evidence for the cognitive benefits of amplification [9,13]. Mulrow et al. conducted one of the largest early randomised controlled trials examining hearing aids and found substantial cognitive improvements at 4 months compared to wait-list controls, particularly in the attention and working memory domains [39]. The ACHIEVE trial [11] (n = 977, 3-year follow-up) represents the largest randomised controlled trial to date examining hearing intervention effects on cognition. The primary intention-to-treat analysis found no significant difference in cognitive decline between intervention and control groups in the full sample. However, a pre-specified subgroup analysis of high-risk participants (n = 238 from the ARIC cohort) demonstrated a 48% reduction in the cognitive decline rate with hearing intervention compared to the control [34]. These findings are consistent with those of Maharani et al., who analysed data from the Health and Retirement Study (n = 2040) and found that hearing aid use was associated with more favourable cognitive trajectories over an 18-year follow-up period [6].

Importantly, not all intervention studies have reported consistent cognitive gains of hearing aid usage, and, to give a balanced view, null/negative results should be considered along with positive results. Null or domain-specific effects were found in some studies. Dawes et al. [12] found that, after hearing aid provision, measures of episodic memory did not demonstrate the same level of improvement as those of executive function and processing speed, indicating cognitive-domain specificity in response. A meta-analysis by Yang et al. [42], who specifically evaluated randomised controlled trials, reported that hearing aid use did not have substantial global positive effects on cognition among individuals without dementia or with Alzheimer’s disease, and showed very modest domain-specific effects on executive function. These null and mixed results from well-designed studies are important to consider in balance with positive observational findings, as they indicate that the cognitive benefits of amplification may be more limited than initially suggested by early observational research. It is conceivable that publication bias towards positive results could have inflated effect estimates in the previous literature and narrative syntheses.

The gap between observational and randomised trial evidence deserves explicit acknowledgment. Despite large-cohort studies, such as the ones by Maharani et al. [6] and Amieva et al. [10] systematically reviewed, and observational studies almost uniformly finding links between hearing aid use and less cognitive decline, results have been more mixed in randomised controlled trials. This pattern suggests a potential for confounding by indication in observational studies: individuals who opt to get and use hearing aids may systematically differ from non-users in terms of education, socioeconomic status, health literacy, access to medical care, and general health-seeking behaviour overall, factors that independently predict better cognitive outcomes. The ACHIEVE trial [11] somewhat mitigates this concern by including randomisation, but its result of cognitive benefits in the high-risk subgroup only, with minimal to no effects in the whole sample and healthier participants, indicates that, even under highly controlled experimental conditions, cognitive benefits are not guaranteed. Randomised studies with a pre-defined subgroup analysis are needed in the future to determine which subpopulation of patients will benefit from hearing intervention regarding cognitive effects.

The cognitive benefits of hearing aid use are closely related to patterns of device utilisation. Sarant et al. found a positive dose–response relationship between daily hearing aid use and cognitive outcomes, with participants using devices for more than 4 h daily showing up to twice the improvement compared to those using devices for fewer than 4 h [58]. Furthermore, cumulative exposure appears to be an important factor, as indicated by Maharani et al. [17], who found that consistent use over time (measured as consecutive time points of use over 5 years) was associated with significantly greater cognitive benefit compared to inconsistent use, suggesting that regular auditory stimulation may be necessary for maintaining neural pathways and cognitive function.

### 4.8. Barriers to Timely Hearing Aid Adoption

Several obstacles impede early hearing aid fitting, resulting in an average delay of 7 to 10 years between identification of hearing problems and treatment initiation. Structural barriers within the healthcare system represent significant impediments to early intervention. Reed et al. found that few individuals with hearing loss were referred for audiological assessment by primary care providers, representing a substantial gap in routine healthcare provision [28]. This system-level bottleneck is compounded by inefficiencies in the care pathway; Arnold et al. found that individuals requiring two or more referrals before reaching audiological services were 2.4 times less likely to be fitted with hearing aids compared to those with more direct pathways [59]. Patient beliefs and attitudes constitute additional significant barriers to early treatment. A review of the literature involving 15,392 adults with self-reported hearing loss identified stigma, denial of hearing problems, and concerns about appearance as the most commonly reported barriers to hearing aid use [60,61]. Wallhagen reported that individuals with mild-to-moderate hearing loss were more likely than those with more severe impairment to attribute delays in hearing aid adoption to concerns about stigma. This perception may partly explain why uptake is often postponed in this population, despite the fact that earlier use of hearing technology could confer the greatest benefit [62]. Economic and logistical factors further impede timely intervention. Simpson et al. examined data from 30 countries and found that patient cost burden was the most significant predictor of hearing aid utilisation rates, with each additional 10% increase in out-of-pocket costs associated with an approximately 15% reduction in utilisation [24]. In the United States specifically, Mahmoudi et al. found that the lack of Medicare coverage for hearing aid expenses was linked to substantial disparities: higher-income beneficiaries were 66% more likely to use hearing aids than their lower-income counterparts [25]. These economic barriers disproportionately affect disadvantaged populations, as described by Nieman et al., who found that socioeconomic factors compounded by geographic barriers resulted in significant hearing healthcare access inequalities, with minority and rural individuals experiencing on average a 2.2-year longer treatment delay compared to their counterparts [61].

### 4.9. Clinical Implications and Best Practices

The growing body of literature on hearing loss, cognition, and intervention timing has several important clinical implications for optimising patient outcomes. Early identification of hearing loss is crucial for preventing what may become a downward spiral of consequences associated with untreated hearing impairment. Yueh et al. demonstrated that universal screening in primary care using an age threshold of 50 years or older resulted in far earlier intervention compared to symptom-based approaches [41]. To advance this approach, Livingston et al. proposed integrating routine cognitive assessment into hearing screening programmes for older adults, on the premise that identifying early age-related cognitive vulnerability may allow intervention at a stage when reversibility is greatest [63]. In line with this perspective, Lin et al. suggested that such screening strategies should incorporate both audiometric measures and functional communication assessments, as evidence indicates that clinically meaningful deficits often emerge before conventional intervention thresholds are reached [64]. Additionally, for individuals with occupational noise exposure who may not consistently use hearing protection, screening remains important since hearing loss may manifest at an early age in such populations [60]. Combined cognitive and hearing intervention approaches are of particular interest from a whole-person care perspective. Dawes et al. reported that cognitive intervention combined with hearing aid fitting resulted in significantly greater benefit for both listening function and attentional performance compared to standard audiological care alone [65]. Similarly, Saunders et al. reported a 32% increase in hearing aid adherence when group therapy addressing the psychosocial aspects of hearing loss was used compared to technology-only interventions, along with a 24% improvement in cognitive outcomes [13]. Patient education and support at the time of amplification transition are essential for influencing long-term outcomes and adherence [66]. Ferguson et al. demonstrated that a structured self-management programme incorporating realistic goal setting and problem solving doubled hearing aid use during the critical first three months compared to traditional fitting processes [40]. Hickson et al. found greater hearing aid use among participants who received increased partner involvement in rehabilitation, emphasising the importance of considering the social context of hearing loss [67]. Together, these findings suggest that optimal intervention is multifaceted and that clinical referral should address not only auditory function but also the cognitive, psychological, and social aspects of hearing health throughout the patient’s engagement with services.

Meta-analyses should be interpreted with caution, as there are limitations in the quality of evidence underpinning them. The meta-analysis conducted by Yeo et al. [43] showed a small (~3%) nominal change in cognitive test scores and a 19% significant decrease in the hazard of long-term cognitive decline with hearing restorative devices. But it is worth noting, as the authors acknowledged, that much of this evidence comes from observational studies and not randomised controlled trials—which greatly hampers its use for making causal claims. The small effect sizes with broad confidence intervals in various pooled analyses are also suggestive of a substantial statistical uncertainty. Additionally, the risk of publication bias, the increased likelihood of studies with positive or significant findings being published compared with those with null results, could systematically inflate pooled estimates. Clinicians should aim to provide patients with realistic, evidence-based expectations: hearing aids are consistently effective at enhancing communication function and hearing-specific quality of life [13,39], but their effects on cognitive decline prevention remain uncertain and appear to be most relevant to individuals with elevated baseline dementia risk factors [11,34]. Exaggerated claims of cognitive benefits may create unrealistic patient expectations and disillusionment if cognitive decline progresses despite eventual consistent hearing aid use.

As a narrative review conducted in accordance with SANRA guidelines, this study synthesises and interprets findings from the existing literature rather than providing primary data. While narrative reviews do not employ the exhaustive and reproducible search strategies characteristic of systematic reviews, we have endeavoured to provide transparency regarding our literature search approach and to present a balanced synthesis of the available evidence.

## 5. Conclusions

The reviewed studies consistently indicate that hearing loss is significantly associated with cognitive impairment and psychosocial decline through numerous interrelated mechanisms. The timing of hearing aid intervention appears to be a key consideration in outcomes across these areas, with early intervention related to better cognitive health, better psychological adaptation, and greater positive neuroplastic changes. This relationship is also proposed to be due to the cognitive load hypothesis, stating that, in individuals with hearing loss, cognitive resources are diverted from processing incoming auditory information to support effortful listening at the expense of other cognitive functions. Such a cascade can be disrupted through well-timed amplification, thereby reducing the listening effort required and safeguarding cognitive resources.

But the public health evidence is riven by some critical paradoxes that must be recognised. Randomised controlled trial evidence supports the notion that hearing aid use provides cognitive benefit in high-risk subgroups, but it does not support the notion that it provides universal cognitive protection [68,69]. The overall null finding of the ACHIEVE trial, the lack of statistically significant cognitive changes in the well-powered Beaver Dam Offspring Study analysis by Dawes et al. [12], and the non-significant results from the extensive CONSTANCES cohort of Grenier et al. [16] all point to a more nuanced view than the one initially offered by observational studies. This kind of risk–benefit profile in the domain is also further complicated by the particular, feature-specific pattern of benefit seen with use in systematic reviews [13,42], where executive function tends to show more persistent gain than attention or episodic memory. The cognitive benefits of hearing aids may be most apparent in those with pre-existing risk factors related to cognitive decline, and the audiologist should manage patients’ expectations accordingly. Although hearing aids are known to have established benefits for communication, social participation, and HL-specific QOL, the contribution of such a device to cognitive preservation may be most pertinent in certain at-risk patient populations rather than in the population of older adults with HL.

Early intervention also seems to forestall the social isolation and the communication frustration that contribute to mood disorders in individuals with hearing impairment. Studies of neuroplasticity offer valuable insights into the timing of these influences, indicating trajectories of age-related neural adaptation and windows of opportunity for intervention. The length of auditory deprivation has systematically been shown to be a major predictor of outcome, with increasing duration of untreated auditory deprivation being related to decreased potential for neural reorganisation and functional recovery. This is a strong indication that the tradition of late intervention—typically 7–10 years post-identification of hearing loss—may substantially undercut possible gains. Even with evidence supporting early amplification intervention, barriers to early adoption of hearing aids persist. Removing these barriers will require coordinated efforts across the healthcare ecosystem, better practices in screening, integrated cognitive/hearing healthcare, and patient education. Financial access is a major issue, demonstrating the need for policy changes in hearing healthcare. The next phase of investigations should involve prospective trials investigating the ideal timing for intervention and the age group(s) and hearing loss groups(s) that will experience maximum benefits. Furthermore, research is needed on personalised interventions that consider differences between individuals in cognitive reserve, psychological resilience, and neural plasticity, thereby also revealing who is most at risk of the negative effects of a late intervention. By addressing the time-varying natural history of hearing loss sequelae while also tackling practical impediments to early intervention, clinicians can maximise hearing outcomes and perhaps even reduce the cognitive and psychological impact of music and other sound sources on the everyday listening experience.

## Figures and Tables

**Table 1:** Characteristics of included longitudinal cohort studies (n = 20).

Study (Reference)	Design	Sample Size	Population	Age (Years)	Follow-Up Duration	Primary Outcomes	Definition of Early vs. Delayed Intervention
Lin et al., 2013 [3]	Prospective cohort	1984	Community-dwelling older adults, USA	≥70	12 years	Cognitive decline (3MS, DSST)	Not applicable (observational exposure)
Amieva et al., 2015 [10]	Prospective cohort	3670	Community-dwelling, France (PAQUID)	≥65	25 years	Cognitive trajectory (MMSE), dementia	Early: HA fitted ≤3 years from diagnosis; delayed: >3 years
Amieva et al., 2018 [5]	Prospective cohort	3777	Community-dwelling, France (PAQUID)	≥65	25 years	Depression, disability, dementia, mortality	Not specified for timing
Maharani et al., 2018 [6]	Prospective cohort	2040	Community-dwelling, USA (HRS)	≥50	18 years	Episodic memory decline	Consistent use vs. inconsistent use over time
Maharani et al., 2019 [17]	Prospective cohort	8340	Community-dwelling, UK (ELSA)	≥50	10 years	Cognitive function, loneliness, social isolation	Not specified for timing
Deal et al., 2015 [18]	Prospective cohort	253	Community-dwelling, USA (ARIC)	67–84	20 years	Cognitive decline (neurocognitive battery)	Earlier onset (<65 years) vs. later onset (≥75 years)
Deal et al., 2016 [19]	Prospective cohort	1889	Community-dwelling, USA (Health ABC)	70–79	9 years	Incident dementia, cognitive decline	Not applicable (observational exposure)
Golub et al., 2017 [20]	Prospective cohort	1881	Multiethnic cohort, USA	≥65	25 years	Incident dementia	Not applicable (dose–response analysis)
Dawes et al., 2015 [12]	Prospective cohort	666	Community-dwelling, USA (BOSS)	21–84	11 years	Cognitive function, mental health, social engagement, mortality	Not specified for timing
Cosh et al., 2018 [21]	Prospective cohort	3154	Community-dwelling, Norway (Tromsø)	≥55	6 years	Depression, anxiety	Not applicable (observational exposure)
Contrera et al., 2016 [22]	Prospective cohort	113	Cochlear implant candidates, USA	≥50	12 months	Quality of life (HUI3)	Not specified for timing
Contrera et al., 2017 [14]	Prospective cohort	113	Cochlear implant recipients, USA	≥50	12 months	Loneliness (UCLA scale)	Not specified for timing
Choi et al., 2016 [15]	Prospective cohort	154	HA/CI recipients, USA	≥50	12 months	Depressive symptoms (GDS)	Not specified for timing
Brewster et al., 2018 [23]	Prospective cohort	8529	Community-dwelling, USA (NHANES)	≥60	Cross-sectional with age stratification	Depression (PHQ-9)	Early: HA within 2 years; delayed: >2 years
Simpson et al., 2019 [24]	Prospective cohort	493	Adults with hearing loss, USA	48–92	15 years	Time to hearing aid adoption	Candidacy to adoption interval measured
Mahmoudi et al., 2018 [25]	Retrospective cohort	934,290	Medicare beneficiaries, USA	≥66	3 years	Healthcare utilisation, costs	HA users vs. non-users
Mahmoudi et al., 2019 [26]	Retrospective cohort	114,862	Medicare beneficiaries, USA	≥66	5 years	Diagnosed dementia, depression, falls	HA users vs. non-users
Pronk et al., 2011 [27]	Prospective cohort	1511	Community-dwelling, Netherlands (LASA)	60–90	4 years	Loneliness, depression	Not specified for timing
Reed et al., 2019 [28]	Retrospective cohort	77,000+	Administrative data, USA	Various	10 years	Healthcare costs, utilisation	Treated vs. untreated HL
Lin et al., 2011 [29]	Prospective cohort	347	Community-dwelling, USA (BLSA)	60–80	6 years	Cognitive function (neuropsychological battery)	Not applicable (observational exposure)

**Table 3:** Summary of cognitive and psychological outcomes by study type and domain.

Study (Reference)	Global Cognitive Function	Executive Function	Episodic Memory	Attention/Processing Speed	Depression/Anxiety	Quality of Life/Social Function
**Randomised Controlled Trials**						
Lin et al., 2023 (ACHIEVE) [11]	NULL (full sample)/POSITIVE (high-risk subgroup)	Not separately reported	Not separately reported	Not separately reported	NULL	POSITIVE
Mulrow et al., 1990 [39]	POSITIVE	Not separately reported	Not separately reported	POSITIVE	POSITIVE	POSITIVE
Sarant et al., 2020 [9]	POSITIVE	POSITIVE	INCONCLUSIVE	POSITIVE	Not assessed	Not assessed
Ferguson et al., 2016 [40]	Not assessed	Not assessed	Not assessed	Not assessed	Not assessed	POSITIVE (HA use)
Doherty & Desjardins, 2015 [34]	Not assessed	Not assessed	Not assessed	POSITIVE (younger); NULL (older)	Not assessed	Not assessed
Yueh et al., 2010 [41]	Not assessed	Not assessed	Not assessed	Not assessed	Not assessed	POSITIVE (QoL)
**Longitudinal Cohort Studies**						
Lin et al., 2013 [3]	NEGATIVE (HL as risk factor)	Not separately reported	Not separately reported	Not separately reported	Not assessed	Not assessed
Maharani et al., 2018 [6]	POSITIVE	Not separately reported	POSITIVE	Not separately reported	Not assessed	Not assessed
Amieva et al., 2015 [10]	POSITIVE (early); NULL (delayed)	Not separately reported	Not separately reported	Not separately reported	Not assessed	Not assessed
Amieva et al., 2018 [5]	Not separately reported	Not separately reported	Not separately reported	Not separately reported	POSITIVE (association)	POSITIVE
Dawes et al., 2015 [12]	NULL	Not separately reported	NULL	NULL	NULL	POSITIVE (hearing handicap only)
Deal et al., 2016 [19]	NEGATIVE (HL as risk factor)	Not separately reported	Not separately reported	Not separately reported	Not assessed	Not assessed
Golub et al., 2017 [20]	NEGATIVE (HL as risk factor)	Not separately reported	Not separately reported	Not separately reported	Not assessed	Not assessed
Mahmoudi et al., 2019 [26]	POSITIVE	Not assessed	Not assessed	Not assessed	POSITIVE	Not assessed
Grenier et al., 2024 [16]	NULL (overall); POSITIVE (with depression)	Not separately reported	Not separately reported	Not separately reported	Interaction effect only	Not assessed
Contrera et al., 2016 [22]	Not assessed	Not assessed	Not assessed	Not assessed	Not assessed	POSITIVE
Contrera et al., 2017 [14]	Not assessed	Not assessed	Not assessed	Not assessed	POSITIVE	POSITIVE
Choi et al., 2016 [15]	Not assessed	Not assessed	Not assessed	Not assessed	POSITIVE	Not assessed
Brewster et al., 2018 [23]	Not assessed	Not assessed	Not assessed	Not assessed	POSITIVE (early); NULL (delayed)	Not assessed
Cosh et al., 2018 [21]	Not assessed	Not assessed	Not assessed	Not assessed	POSITIVE (association)	Not assessed
Pronk et al., 2011 [27]	Not assessed	Not assessed	Not assessed	Not assessed	POSITIVE	POSITIVE
**Systematic Reviews/Meta-Analyses**						
Sanders et al., 2021 [13]	INCONCLUSIVE	POSITIVE (6/11 studies)	INCONCLUSIVE (3/5 studies positive)	NULL (8/9 studies no effect)	Not assessed	Not assessed
Yang et al., 2022 [42]	NULL	POSITIVE (modest)	NULL	NULL	Not assessed	Not assessed
Yeo et al., 2023 [43]	POSITIVE (~3% improvement; mostly observational)	Not separately reported	Not separately reported	Not separately reported	Not assessed	Not assessed
Loughrey et al., 2018 [44]	POSITIVE (association with HL)	POSITIVE	POSITIVE	Not separately reported	Not assessed	Not assessed
Thomson et al., 2017 [33]	POSITIVE (HL as risk factor for dementia)	Not separately reported	Not separately reported	Not separately reported	Not assessed	Not assessed
Lawrence et al., 2020 [35]	Not assessed	Not assessed	Not assessed	Not assessed	POSITIVE (OR 1.47)	Not assessed
**Neuroimaging/Neuroplasticity Studies**						
Glick & Sharma, 2020 [7]	POSITIVE	POSITIVE	Not assessed	POSITIVE	Not assessed	Not assessed
Campbell & Sharma, 2014 [32]	Not assessed (neuroimaging only)	—	—	—	—	—
Alain et al., 2014 [30]	POSITIVE (short deprivation); NULL (long deprivation)	—	—	—	—	—
Peelle et al., 2011 [8]	Not assessed (neuroimaging only)	—	—	—	—	—

## Data Availability

No new data were created or analyzed in this study. Data sharing is not applicable to this article.

## Supplementary Materials

The following supporting information can be downloaded at: https://www.mdpi.com/article/10.3390/audiolres16020032/s1, Table S1: Characteristics of included cross-sectional, review, and conceptual studies (n = 8).

## Abbreviations

The following abbreviations are used in this manuscript:

3MS	Modified Mini-Mental State Examination
ABR	Auditory Brainstem Response
BTE	Behind-the-Ear (Hearing Aid)
CAEPs	Cortical Auditory Evoked Potentials
CIC	Completely-in-the-Canal (Hearing Aid)
DSST	Digit Symbol Substitution Test
fMRI	Functional Magnetic Resonance Imaging
FUEL	Framework for Understanding Effortful Listening
GDS	Geriatric Depression Scale
HA	Hearing Aid
HADS	Hospital Anxiety and Depression Scale
HL	Hearing Loss
HUI3	Health Utilities Index Mark 3
MMSE	Mini-Mental State Examination
PHQ-9	Patient Health Questionnaire-9
QoL	Quality of Life
