# Supplementary material for: Mood and Cognitive Disorders Following Hearing Loss: Impact of Hearing Aid Timing
Source: Audiol Res. 2026 Feb 26;16(2):32. doi: 10.3390/audiolres16020032 (PMC13010665; doi:10.3390/audiolres16020032)
Supplement: Supplementary file 1 [file audiolres-16-00032-s001.zip › audiolres-4018747-supplementary.pdf]

Table S1. Characteristics of Included Cross-Sectional, Review, and Conceptual Studies (n = 8).

Study (Reference)	Design	Sample Size	Population	Age (years)	Primary Outcomes	Notes
Peelle et al., 2011 [8]	Cross-sectional neuroimaging	18	Adults with varying hearing, USA	60–77	Neural activity (fMRI)	Examined neural correlates of hearing loss
Arnold et al., 2019 [60]	Cross-sectional	2,563	Hispanic adults, USA (HCHS/SOL)	≥50	HA prevalence, factors for use	Identified demographic predictors of HA use
Nieman et al., 2016 [62]	Cross-sectional	1,636	Community-dwelling, USA (NHANES)	≥70	Disparities in hearing healthcare	Treatment delay measured by demographics
Bisgaard & Ruf, 2017 [46]	Cross-sectional surveys (pooled)	~75,000	European population (EuroTrak)	Various	HA adoption, satisfaction	Surveys conducted 2009–2015
Grenier et al., 2024 [16]	Cross-sectional with longitudinal elements	62,072	Community-dwelling, France (CONSTANCES)	45–69	Global cognitive impairment	HA users with disabling HL vs. non-users
Slade et al., 2020 [48]	Narrative review	Not applicable	Various	Various	Brain structure/function	Reviewed effects of age-related HL on brain
Pichora-Fuller et al., 2016 [30]	Conceptual framework	Not applicable	Theoretical	Not applicable	Cognitive load (FUEL framework)	Proposed Framework for Understanding Effortful Listening
Wallhagen, 2010 [37]	Cross-sectional/qualitative	Various	Community-dwelling, USA	Various	Stigma, HA adoption	
